# Expression and function of an *Hac1*-regulated multi-copy xylanase gene in *Saccharomyces cerevisiae*

**DOI:** 10.1038/s41598-020-68570-6

**Published:** 2020-07-15

**Authors:** Changjie Bao, Jiping Li, Huan Chen, Yang Sun, Gang Wang, Guang Chen, Sitong Zhang

**Affiliations:** 1 College of Life Sciences, Jilin Agricultural University, Changchun, China; 2 Key Laboratory of Straw Biology and Utilization, The Ministry of Education, Changchun, China; 3 Institute of Antler Science and Product Technology, Changchun Sci-Tech University, Changchun, China

**Keywords:** Biophysical methods, Microbiology techniques, Genetic engineering

## Abstract

*Saccharomyces cerevisiae*-based expression systems, which rely on safe, food-grade strains, are low cost, simple to operate, and can be used for large-scale fermentation. However, low levels of foreign protein expression by *S. cerevisiae* have limited their widespread application. The ability of the endoplasmic reticulum (ER) to fold and process foreign proteins is an important factor restricting the expression of foreign proteins. In the current study, the effects of transcription factor Hac1p, which is involved in the unfolded protein response pathway, on *S. cerevisiae*-based expression of xylanase gene *xynB* from *Aspergillus niger* were examined. Overlap extension polymerase chain reaction (PCR), rDNA integration and droplet digital PCR technology were used to generate a *S. cerevisiae* strain (S8) containing eight copies of *xynB*, allowing high-yield secretory expression of xylanase. The effects of subsequent overexpression of *HAC1* in strain S8 on the expression of genes associated with protein folding in the ER were then examined using the GeXP system. Results confirmed the constitutive secretory expression of the multiple copies of *xynB* following rDNA-based integration of the expression cassette, with a maximum xylanase yield of 325 U/mL. However, overexpression of *HAC1* further improved xylanase production by strain S8, resulting in a yield of 381 U/mL.

## Introduction

Xylanase, a microbial hydrolase used to hydrolyse xylan into xylooligosaccharide and xylose, is frequently used in the energy industry, but is also widely applied in fields ranging from livestock feed and human food production to papermaking^1,2^. At present, the main factor limiting the production and application of xylanase is the lack of high-yield bacterial and fungal strains that can be adapted to large-scale production. Therefore, it is imperative to research and develop new high-yield xylanase-producing strains^3^.

Approved by the Food and Drug Administration of the United States as a safe species, *Saccharomyces cerevisiae* is an ideal host for the expression of enzymes used in food and feed production^4^. With its well defined biological and genetic background, *S. cerevisiae* boasts the advantageous features of prokaryotes, including fast growth and reproduction and simple genetic manipulation procedures, as well as the post-translational modification of heterologous proteins that is common to eukaryotes. As such, *S. cerevisiae* has been widely studied and used for the expression of heterologous genes^5,6^. Ribosomal DNA (rDNA), coding for ribosomal RNA, consists of a tandemly repeated unit containing both transcribed and untranscribed regions. Chromosome 12 of the *S. cerevisiae* genome contains approximately 100–140 repeating rDNA units; thus, if the rDNA sequence is used as an integration site, 100–140 copies of a target gene should theoretically be obtained^7,8^. Dosage effects mean that highly efficient gene expression is achieved in *S. cerevisiae* following rDNA-mediated integration of heterologous genes.

The unfolded protein response (UPR) signalling pathway, an endoplasmic reticulum (ER) stress response pathway, acts as a quality control mechanism in the synthesis of secreted or membrane-localised proteins, ensuring proper protein folding or degradation^9^. Multiple proteins are involved in the signalling pathway, including Hac1p, Cpr5p, Cne1p, Ero1p, Kar2p, Pdi1p and Sec53p. As a transcription factor, Hac1p is a key regulator of the UPR pathway. Cpr5p is a cyclophilin, the main functions of which are to affect peptidyl-prolyl *cis*-*trans* isomerase, participate in post-translational modification of proteins and maintain the homeostasis of intracellular metal ions. Cne1p, a calcium-binding protein, acts as a molecular chaperone in the ER and participates in protein folding and glycosylation modification. Protein disulphide-isomerase (PDI) oxidase Ero1p controls the folding of oxidised proteins in the ER, while Kar2p is a Bip-binding protein that transports proteins to the ER with the help of an ATPase. In addition, Kar2p acts as a molecular chaperone, assisting with protein folding. Pdi1p is a multifunctional molecular chaperone in the ER and plays a major role in the formation of disulphide bonds. Finally, Sec53p, a phosphomannomutase, is involved in the synthesis of GDP-mannose and mannose 6-phosphate, which are required for the folding and glycosylation of secretory proteins in the ER lumen. The genes coding for each of these proteins are likely to be affected by Hac1p-mediated activation of the UPR pathway, resulting in both short- and long-term physiological effects^9,10^.

In the current study, constitutive promoter *PGK1p*, the α-factor signal peptide, the *xynB* mature peptide sequence and *CYC1T* were ligated to obtain expression cassette PαXC. An rDNA integration method was then used to construct multi-copy xylanase-expressing *S. cerevisiae* strains to investigate the relationship between copy number and xylanase expression via droplet digital polymerase chain reaction (ddPCR) analysis. By overexpressing *HAC1* in the strains, the multi-copy xylanase expression was optimised. The GeXP genetic analysis system was then used to examine the impact of *HAC1* overexpression on the expression of genes associated with protein folding in the ER, providing a theoretical basis for reconstructing and modifying *S. cerevisiae* strains used in xylanase production.

## Materials and methods

### Strains, plasmids, media and culture conditions

The strains and plasmids used in this study are described in Table 1. *Escherichia coli* DH5α was used as the host for gene cloning experiments. *S. cerevisiae* INVSc1 (Invitrogen, Carlsbad, CA, USA) was selected for use in this study because it shows high level expression of heterologous proteins. Strain INVSc1 was used as the host for *xynB* expression and as a template to amplify the *PGK1p* sequence (850 bp, GenBank accession number: AH001380), the rDNA sequence (2,300 bp, GenBank accession number: BK006945.2), and *HAC1* (717 bp, GenBank accession number: NM_001179935). *Aspergillus niger* strain CICC2462 was used as a template to amplify *xynB* (984 bp, GenBank accession number: FJ986225.1), while plasmids pPic9k and pSH65 were used as templates to amplify the α-factor secretion signal sequence (255 bp, GenBank accession number: KM032189) and *CYC1T* (254 bp, GenBank accession number: AF298780.1), respectively. *S. cerevisiae* INVSc1[pYES2-*xynB*], a single-copy xylanase *S. cerevisiae* expression strain, was used to express xylanase under galactose induction^11^.

**Table 1:** Strains and plasmids used in this study.

	Characteristics	Sources
**Strains**		
E. coli DH5*α*	Host of gene cloning	Takara, Japan
*Aspergillus niger CICC2462 strain*	Used to clone *xynB* gene	China Center of Industrial Culture Collection
*S. cerevisiae* INVSc1 (Wild type *S. cerevisiae,*Hereinafter referred to as S0)	Expression host of *MATa*, *his3*, *leu2, trp*, *ura3*	Invitrogen, America
*S.cerevisiae* INVSc1[pYES2*-xynB*]	Galactose induced xylanase *Saccharomyces cerevisiae* expression	Lan et al.^11^
(Hereinafter referred to as S1)	Constitutive single-copy expression of xylanase gene *Saccharomyces cerevisiae*	This study
*S. cerevisiae* INVSc1[pYES2-N × *PαXC*](Hereinafter referred to as SN)	Constitutive multi-copy expression of xylanase gene *Saccharomyces cerevisiae*	This study
*S. cerevisiae* INVSc1[*Hac1*](Hereinafter referred to as S0-H)	*Hac1* gene overexpression of wild-type *Saccharomyces cerevisiae*	This study
*S. cerevisiae* INVSc1[pYES2-*PαXC*-*Hac1*](Hereinafter referred to as S1-H)	*Hac1* gene overexpression of single-copy xylanase gene *Saccharomyces cerevisiae*	This study
*S. cerevisiae* INVSc1[pYES2-N × *PαXC*-*Hac1*](Hereinafter referred to as SN–H)	*Hac1* gene overexpression of multi-copy xylanase gene *Saccharomyces cerevisiae*	This study
**Plasmid**		
pYES2	Expression host of *ura3 Saccharomyces cerevisiae*	Invitrogen, America
pYES6	Host of Blasticidin resistance and the expression *Saccharomyces cerevisiae ura3*	Invitrogen, America
pPIC9K	Applied to amplify signal peptide *α-factor* sequence	China Center for Type Culture Collection
pMD19-T	Host of gene cloning	Takara, Japan
pSH65	Used to amplify *CYC1T*	China Center for Type Culture Collection
pYES2-*PαXC*	Expression host of constitutive single-copy xylanase	This study
pYES2-*PαXC-rDNA*	Expression host of constitutive multi-copy xylanase	This study
pYES6-*PGK*-*HAC1*	*Hac1* gene overexpression host	This study

*E. coli* DH5α was cultured in Luria–Bertani medium at 37 °C. *S. cerevisiae* INVSc1 culture and fermentation were performed in YPD medium containing 20 g/L peptone, 10 g/L yeast extract, and 10 g/L glucose at 30 °C. Synthetic complete medium lacking uracil (SC-URA) (6.7 g/L yeast nitrogen base, 20 g/L glucose, 20 g/L agar, 1 g/L (adenine, arginine, cysteine, leucine, lysine, threonine, tryptophan), and 0.5 g/L (aspartic acid, histidine, isoleucine, methionine, phenylalanine, proline, serine, tyrosine, valine); Clontech, Mountainview, CA, USA) was used for the selection of *S. cerevisiae* INVSc1[pYES2-PαXC] recombinants. Xylan-Congo red medium (20 g/L peptone, 10 g/L yeast extract, 10 g/L xylan (Sigma Aldrich, St Louis, MO, USA), 18 g/L agar, and 1% (w/v) Congo red) was used as a xylanase vitality culture medium, with cultures incubated at 30 °C. Galactose medium (20 g/L peptone, 10 g/L yeast extract, and 10 g/L D-galactose) was used for enzymatic fermentation of *S. cerevisiae* INVSc1[pYES2-xynB].

### Construction of the PαXC expression cassette by overlap extension PCR

Following activation, *A. niger* strain CICC2462 was inoculated into liquid fermentation medium and cultivated at 30 °C and 150 rpm for 72 h. Cells were collected by centrifugation and RNA was extracted from the resulting cell pellets using an AXYGEN Total RNA Extraction Kit (Fisher Scientific, Waltham, MA, USA). A PrimeScript One-Step RT-PCR Kit (Takara Bio, Kusatsu, Japan) was then used to generate cDNA, which was used as template to amplify *xynB*.

*S. cerevisiae* INVSc1 genomic DNA was used as a template to amplify the phosphoglycerate kinase (*PGK1*) promoter sequence, while plasmids pPIC9K and pSH65 were used as templates to amplify the α-factor secretion signal sequence and the *CYC1T* sequence, respectively. Overlap extension PCR was used to ligate the four sequences, generating a *PGK1p*-α-factor-*xynB-CYC1T* expression cassette designated PαXC. *EcoR*I and *Xba*I recognition sites were introduced upstream of *PGK1p* and downstream of *CYC1T*, respectively. Primers used for all amplifications are listed in Table 2.

**Table 2:** Primers used in this study.

Prime	Sequence (5′–3′)	Annotation
*xynB*-F	ATGGTTCAGATCAAGGTAGCTG	Used to amplify *xynB* gene
*xynB*-R	CTAGAGAGCATTTGCGATAGC	Used to amplify *xynB* gene
*PGK1*-F	CCGGAATTCAGCTTTCTAACTGATCTATCCAAAAC	*EcoR*I was used to amplify *PGK1* sequence
*PGK1*-R	GCTGCCTTGATCTGAACCATTGTTTTATATTTGTTGTAAAAAGTAGA	Used to amplify *PGK1* sequence
*α-factor*-F	TCTACTTTTTACAACAAATATAAAACAATGAGATTTCCTTCAATTTTTACTGC	Used to amplify *α-factor* signal sequence
*α-factor*-R	TGTCGATGCTCACTGAAGCCTGTCTTTTCTCGAGAGATACCCCTT	Used to amplify *α-factor* sequence
*xynB*-F1	AAGGGGTATCTCTCGAGAAAAGACAGGCTTCAGTGAGCATCGACA	Used to amplify mature peptide *xynB* gene
*xynB*-R1	GCGTGACATAACTAATTACATGACCTAGAGAGCATTTGCGATAGCAGTGT	Used to amplify mature peptide *xynB* gene
*CYC1*-F	ACACTGCTATCGCAAATGCTCTCTAGGTCATGTAATTAGTTATGTCACGC	Used to amplify *CYC1* sequence
*CYC1*-R	TGCTCTAGACGGCCGCAAATTAAAGCCTT	*Xba*I was used to amplify *CYC1* sequence
rDNA-F	ACGTACGTACAACGAACGAGACCTTAACCT	
rDNA-R	ACGTACGTACGGAACCTCTAATCATTCGCT	
*ACT1*-F1	TATCCCCTGCATCCCTATCA	
*ACT1*-R1	CAGGCTTCGTTGCAGATACA	
*ACT1*-probe	HEX-CATCTTCGTTAGCTTCATCCGACGCTA-BHQ	
*xynB*-F2	GCCGTGGACAGTTCTCTTTC	
*xynB*-R2	TCATGACCCCGATGAGTGTA	
*xynB*-probe	FAM-CCTGGTCAACTTTGCCCAGTCTAACAA-BHQ	
*Hac1*-F	CCCGGATCCATGGAAATGACTGATTTTG	
*Hac1*-R	CCCTCTAGATCATGAAGTGATGAAGAAATC	

### Construction of the *S. cerevisiae* INVSc1[pYES2-PαXC-rDNA] recombinant strain

The PαXC expression cassette was ligated into pMD19-T and transformed into electrocompetent *E. coli* DH5α cells before being extracted and confirmed by sequencing. The extracted pMD19-T-PαXC plasmid and vector pYES2 were then digested with *EcoR*I and *Xba*I, respectively, and visualised by agarose gel electrophoresis. The target PαXC fragment was then recovered from the agarose gel and ligated into the linearised pYES2 vector using T4 ligase, generating plasmid pYES2-PαXC. The recombinant plasmid was then transformed into electrocompetent *E. coli* DH5α.

*S. cerevisiae* INVSc1 genomic DNA was used as a template to amplify the 2,300-bp core sequence of the rDNA unit using primers rDNA-F and rDNA-R, both of which introduced *SnaB*I restriction enzyme recognition sites into the resulting DNA fragment. pYES2-PαXC and pMD19-T-rDNA (constructed by TA cloning) were then digested with *Sna*BI and the target fragments recovered by gel purification. T4 ligase was used to ligate the target fragments, generating recombinant expression vector pYES2-PαXC*-*rDNA. pYES2-PαXC*-*rDNA was then linearised at the rDNA locus using *Sph*I, and the LiAc/ssDNA method^12,13^ was used to transform the linearised vector into electrocompetent *S. cerevisiae* INVSc1. Recombinants were selected by culture on SC-URA medium for 3–5 days.

### Determination of transformant copy number using ddPCR analysis

ddPCR analysis is a recently developed absolute gene quantification method that works by amplifying a single molecule by means of extreme dilution. It can be used to determine the original concentration of a sample by applying end-point PCR analysis and Poisson distribution. The method displays high levels of accuracy and reproducibility, ensuring absolute quantification^14,15^.

In preparation for ddPCR analysis, transformants were inoculated into YPD medium and cells were collected after 48 h of cultivation. A yeast genome extraction kit (Omega Bio-tek, Norcross, GA, USA) was used to extract genomic DNA for use as template for ddPCR. The QX200 ddPCR System (Bio-Rad Laboratories, Hercules, CA, USA) was then used to identify *xynB* gene copy number using the following primers and probes: *ACT1*-F1, *ACT1*-R1 and *ACT1*-probe; *xynB*-F2, *xynB*-R2 and *xynB*-probe. Reference gene *ACT1* was fluorescently labelled with HEX, while target gene *xynB* was labelled with FAM. ddPCR assays were carried out in 20-μL reaction volumes containing 10 μL of 2 × ddPCR Master Mix, 1 μL each of 10 μmol/L forward and reverse primers, 0.5 μL of probe and 2 μL of DNA template. To generate droplets, specialised droplet generator cartridges and gaskets and a droplet generator were used. For each reaction, 40 μL of reaction mixture and 70 μL of droplet generation oil were added to droplet generator cartridges, covered with specialised gaskets and placed into the droplet generator. A two-step ddPCR amplification protocol was used, consisting of a pre-denaturation step at 94 °C for 10 min, followed by 45 cycles of 94 °C for 15 s, 60 °C for 1 min and 98 °C for 10 min. Each template was assayed in triplicate. Following amplification, 96-well plates were transferred to the reader and analysed using QuantaSoft to obtain the absolute quantification^16^.

### Fermentation of transformants to obtain xylanase

Transformants carrying different numbers of copies of *xynB* were separately inoculated into galactose medium or YPD medium in a fermenter and incubated at 30 °C for 72 h with an agitation speed of 150 rpm. Xylanase activity in the resulting cultures was determined using the reducing sugar assay, as described previously^17^ (Sigma Aldrich). The standard curve of the xylose regression equation was as follows: y = 0.1275x − 0.07, with *R*^2^ = 0.9935. Xylan was purchased from Sigma Aldrich (catalogue number V900513).

### Overexpression of HAC1 in the recombinant xylanase-producing *S. cerevisiae* strains

*S. cerevisiae* INVSc1 genomic DNA was used as the template for amplification of the *PGK1p* sequence using primers *PGK1p*-F1 (CCCAAGCTTCTGCCCCAGGTTCCGTTATT) and *PGK1p*-R1 (CGCGGATCCACCGAAGGCATCGTTGATGT). The resulting amplicon was ligated into pYES6 at the *Hin*dIII and *Bam*HI restriction sites, generating recombinant plasmid pYES6-*PGK1p*.

*S. cerevisiae* strain INVSc1 was inoculated into YPD broth and incubated in a shaking incubator overnight at 30 °C. Sterilised DDT was then added to a final concentration of 5 mM, and the culture was returned to the incubator for 6 h at 30 °C. Upon DDT induction, a large number of unfolded proteins are accumulated by yeast cells, which induces the UPR, resulting in *HAC1* transcription. RNA was then extracted from the DDT-induced cells and reverse-transcribed into cDNA using a PrimeScript One-Step RT-PCR Kit (Takara Bio). The resulting cDNA was used as a template for amplification of *HAC1* using primers *Hac1*-F and *Hac1*-R.

The *HAC1* amplicon was then ligated into pYES6-*PGK1p* via double-enzyme digestion, and the resulting recombinant plasmid, named pYES6-*PGK1p*-*HAC1*, was confirmed by sequencing. pYES6-*PGK1p*-*HAC1* was then linearised and transformed into various *S. cerevisiae* recombinant strains carrying different numbers of copies of *xynB*. Blasticidin resistance was used as a selective marker for the resulting transformants.

### Detection of expression levels of genes associated with protein folding in the ER using the GeXP system

The GeXP genetic analysis system, developed by Beckman Coulter (Brea, CA, USA), is a new platform for quantitative multi-gene expression analysis and gene expression profiling. The system combines capillary electrophoresis-based separation with highly sensitive laser-induced fluorescence technology to achieve high sensitivity and speed for quantitative analysis of gene expression. Using mRNA as the template, multiple PCR reactions initiated by fluorescently labelled universal primers and specific chimeric primers in the same reaction system allow quantitative analysis of up to 25 reverse-transcribed PCR products. The GeXP systems is an advance over existing chip-based and quantitative PCR technologies, allowing the monitoring of dozens to hundreds of genes and the processing of thousands of samples^18^.

For the GeXP assays, fluorescent labels 5ʹ-aggtgacactatagaata-3ʹ and 5ʹ-gtacgactcactataggg-3ʹ were added to the primers used to amplify the target genes, producing fragments that were 37-bp larger than the original amplicon size. The GeXP detection process includes five steps: 1) design multiplex primer sets using NCBI Primer-BLAST; 2) perform reverse transcription reaction; 3) perform PCR reaction; 4) run PCR products on the GeXP system; and 5) carry out data analysis using the GeXP fragment analysis module, GeXP data tool and GeXP quantitative tool.

*S. cerevisiae* strain S0 (wild-type strain INVSc1) and recombinant strains S0-H (wild-type strain expressing *HAC1*), S1 (single-copy *xynB* strain), S1-H (single-copy *xynB* strain expressing *HAC1*), S8 (strain carrying eight copies of *xynB*), S8-H (strain carrying eight copies of *xynB* and expressing *HAC1*; showed the highest xylanase production), S22 (strain carrying 22 copies of *xynB*; the highest copy-number strain) and S22-H (strain carrying 22 copies of *xynB* and expressing *HAC1*) were individually inoculated into YPD medium and incubated at 30 °C. Samples were collected at 24 h, 48 h and 72 h post-inoculation and subjected to RNA extraction. RNA samples were then reverse-transcribed into cDNA and used as template for PCR assays for GeXP-based analysis of the expression of *CPR5*, *CNE1*, *ERO1*, *KAR2*, *HAC1*, *PDI1* and *SEC53*, all of which are associated with protein folding in the ER. The expression of *xynB* at each of the time points was also assessed by GeXP. The primers and the sizes of the resulting fragments are shown in Table 3.

**Table 3:** Primers and fragment sizes for GeXP assays.

Prime	Sequence (5′–3′)	Fragment length	Testing length
*CPR5*-F	aggtgacactatagaatacaccacaccccaaaccgttg	303	340
*CPR5*-R	gtacgactcactatagggaacgtgcttaccgtccaacca		
*CEN1*-F	gtacgactcactatagggatgcagatttgcagaaatacaaga	306	343
*CEN1*-R	gtacgactcactatagggagcatactaaggcacacttatgcaa		
*ERO1*-F	aggtgacactatagaatatctcaccacaccccaaaccg	313	350
*ERO1*-R	gtacgactcactatagggaccaaagacaacgtgcttaccgt		
*HAC1*-F2	aggtgacactatagaataaagacgcgttgacttgcagc	323	360
*HAC1*-R2	gtacgactcactatagggacagagtgggtctgccaacgg		
*KAR2*-F	aggtgacactatagaataggtaagaaggcctccaagggt	335	372
*KAR2-*R	gtacgactcactatagggacttggtgctggtggaatgcc		
*PDI1*-F	aggtgacactatagaataaccttggcccagatcgactg	345	382
*PDI1*-R	gtacgactcactatagggaatcgtctgcgttttcagcgg		
*SEC53*-F	aggtgacactatagaatagctgcattggttttgtcggt	359	396
*SEC53*-R	gtacgactcactatagggactcaccgagccagttgatga		
*xynB*-F4	aggtgacactatagaatagtcatcggcgaggactacgt	370	407
*xynB*-R4	gtacgactcactatagggacgactccccacacggtgata		

## Results

### Construction of the PαXC expression cassette

Following amplification of the fragments needed to construct the expression vector (*PGK1p*, α-factor signal sequence, *xynB* and *CYC1T*), the optimal conditions for overlap extension PCR were determined. The resulting full-length expression cassette, named PαXC, was 2,365 bp in length and contained loci downstream of *xynB* where two sequences could be ligated.

### Construction of multi-copy xynB *S. cerevisiae* INVSc1[pYES2-PαXC-rDNA] strains

The pYES2-PαXC vector was confirmed by sequencing (Genewiz, Suzhou, China). Following digestion with *Sna*BI, pYES2-PαXC was dephosphorylated and ligated with *Sna*BI-treated rDNA, generating pYES2-PαXC-rDNA. *Sph*I-treated pYES2-PαXC-rDNA, linearised at the rDNA loci, was then transformed into *S. cerevisiae* INVSc1 using the optimised LiAC/ssDNA chemical transformation method. Transformants were selected on SC-URA auxotrophic medium followed by Congo red medium containing xylan as the sole carbohydrate source. The xylanase-producing capacity of the transformants was preliminarily assessed based on the size of the hydrolysis circle surrounding each colony. Differences in the diameters of the hydrolysis circles of the transformants indicated that the recombinant strains contained different numbers of copies of *xynB*.

### Evaluation of the effects of *PGK1p* and the α-factor signal peptide on xylanase expression

**Figure 1:**
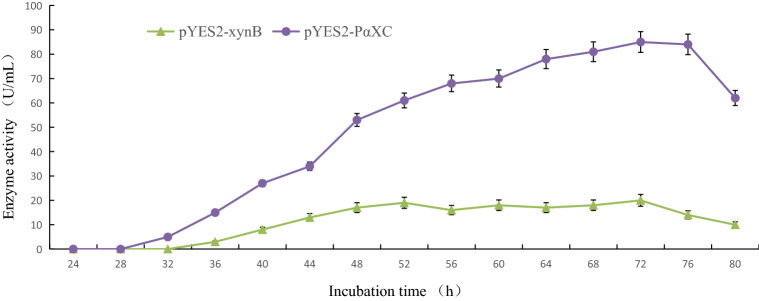
Xylanase activity of *S. cerevisiae* strains INVSc1[pYES2-*xynB*] and INVSc1[pYES2-PαXC]. The effects of the *PGK1p* and the α-factor signal peptide on xylanase production were examined by measuring the xylanase activity of culture supernatants of the two strains.

To achieve constitutive extracellular expression of xylanase, *S. cerevisiae* strain INVSc1[pYES2-*xynB*] was generated and inoculated into YPD medium containing galactose instead of glucose. The selected *S. cerevisiae* INVSc1[pYES2-PαXC] strains were also cultivated for 72 h, with samples collected every 4 h after the first 24 h. The xylanase activity of the culture supernatants from each strain was then examined (Fig. 1). Although maximum xylanase activity was observed for *S. cerevisiae* strain INVSc1[pYES2-PαXC] at 72 h post-inoculation, it showed higher levels of xylanase activity than strain INVSc1[pYES2*-xynB*] throughout the experimental period, indicating that the *PGK1p* and the α-factor signal peptide contributed to xylanase production. It was therefore hypothesised that the *PGK1p* ensured constitutive expression of *xynB*, and that the α-factor signal peptide effectively enabled the extracellular secretion of synthesised xylanase.

### Relationship between *xynB* copy number and xylanase activity as determined by ddPCR

**Figure 2:**
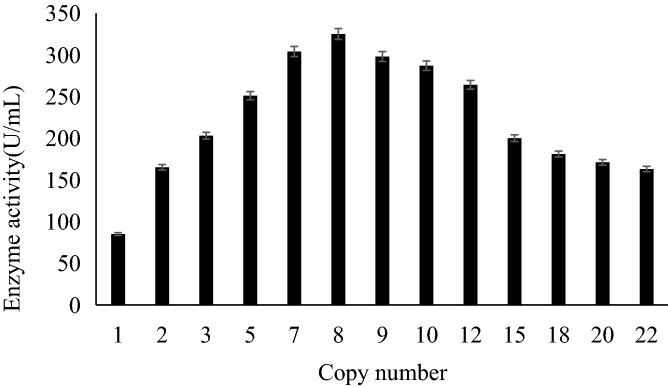
Relationship between xynB copy number and enzyme activity. *Xylanase gene* copy number in each of the different strains is indicated on the x-axis, while the y-axis shows xylanase activity.

DNA was extracted from selected *S. cerevisiae* INVSc1[pYES2*-*PαXC*-*rDNA] transformants, and *ACT1* was used as a reference to determine *xynB* copy number in each strain (Fig. S1 and Table 4). The detected droplet range was between 10,987 and 14,683, and because both values were > 10,000, corresponding to Poisson distribution, it is likely that the results were credible. The identified *xynB* copy numbers were 1.02, 2.11, 2.98, 5.04, 6.95, 8.07, 9.02, 12.09, 15.18, 17.92, 19.98 and 22.00, suggesting that 12 xylanase-expressing recombinant strains, with 1, 2, 3, 5, 7, 8, 9, 12, 15, 18, 20, and 22 copies of *xynB*, respectively, were successfully generated in this study. All of the strains expressed and secreted xylanase during cultivation in YPD medium. The xylanase activity of the fermentation broth from each of the strains was then compared to determine the relationship between enzyme activity and gene copy number (Fig. 2). Results showed that xylanase activity increased with increasing *xynB* copy number. However, while xylanase activity increased almost exponentially up to three copies of *xynB*, activity began to decrease with gene copy numbers greater than eight. Thus, maximum xylanase activity was observed at eight copies of *xynB*, reaching a final yield of 325 U/mL, 4.35-fold higher than the yield of the one-copy transformant. At 18 gene copies, xylanase activity decreased to 181 U/mL, corresponding to only 49% of the yield of the eight-copy strain. Similarly, at 22 copies, the xylanase activity was only 44% of that of the eight-copy strain, but was higher than that of the one-copy strain. These results demonstrated that increases in gene copy number did not necessarily lead to increases in protein expression.

**Table 4:** Analysis of *xynB* copy numbers in *S. cerevisiae* INVSc1[pYES2*-*PαXC-rDNA] transformants by ddPCR.

Sample	Droplets	Concentration (copies·μL^−1^)	Copy number
1	13,682	1578	15.18
2	12,109	1,098	12.09
3	12,784	780	5.04
4	11,099	975	9.02
5	14,683	3,289	22.00
6	11,502	2,698	6.95
7	12,846	3,700	2.98
8	12,198	298	2.11
9	13,108	892	19.98
9	10,987	657	8.07
10	14,542	1884	17.92

### Effects of overexpression of *HAC1* on the expression of xylanase in recombinant *S. cerevisiae* strains

Because *HAC1* contains a 250-bp intron, it is not expressed in normal *S. cerevisiae* cells. However, when improperly folded proteins accumulate in the ER, *HAC1* is spliced, the intron is removed, and active Hac1p is expressed^19^. Therefore, in the current study, improper protein folding was induced in wild-type *S. cerevisiae* using DTT, a protein folding inhibitor, allowing amplification of activated *HAC1*. To generate expression vector pYES6-*PGK1p-HAC1*, the *HAC1* amplicon and constitutive promoter *PGK1p* were ligated into pYES6. pYES6-*PGK1p*-*HAC1* was transformed into wild-type *S. cerevisiae* strain S0 and recombinant strains S1, S8, S9 and S22, generating xylanase- and Hac1p-expressing strains named S0-H, S1-H, S8-H, S9-H and S22-H, respectively.

**Figure 3:**
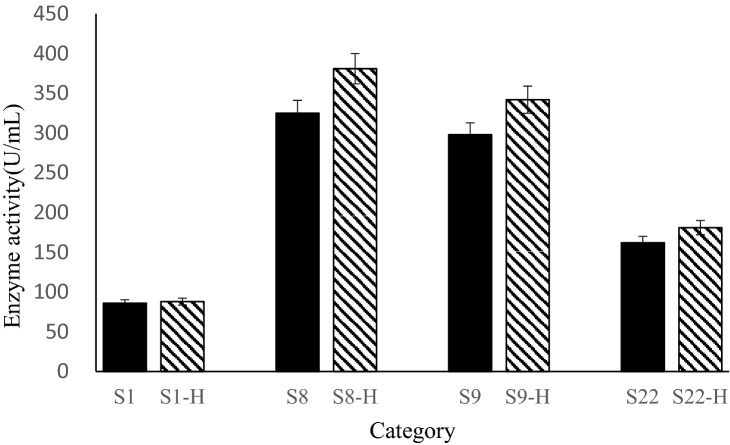
Effects of overexpression of *HAC1* on xylanase enzyme activity. The various tested strains (numbers indicate xynB copy number) are shown on the x-axis, with enzyme activity shown on the y-axis.

Following culture in YPD medium, the xylanase activity of the supernatants of strains S1-H, S8-H, S9-H and S22-H was compared with that of strains S1, S8, S9 and S22 (Fig. 3). Results showed that the xylanase activity of recombinant strain S8-H was 17.2% higher than that of S8, reaching a final yield of 381 U/mL. In addition, the xylanase activities of strains S9-H and S22-H were 14.7% and 11.7% higher than those of strains S9 and S22, respectively, while there was no observable difference between strains S1-H and S1. These results suggested that heterologous protein expression in the single-copy *xynB* strain did not cause ER stress, and that with nine copies of *xynB*, overexpression of *HAC1* did not increase enzyme activity over that of the eight-copy strain. These results suggested that eight copies of *xynB* is optimal for peak xylanase expression in the *S. cerevisiae* expression system.

### Effects of *HAC1* overexpression on the expression of genes associated with protein folding in the ER

#### Specificity of primers used to amplify genes associated with protein folding in the ER

**Figure 4:**
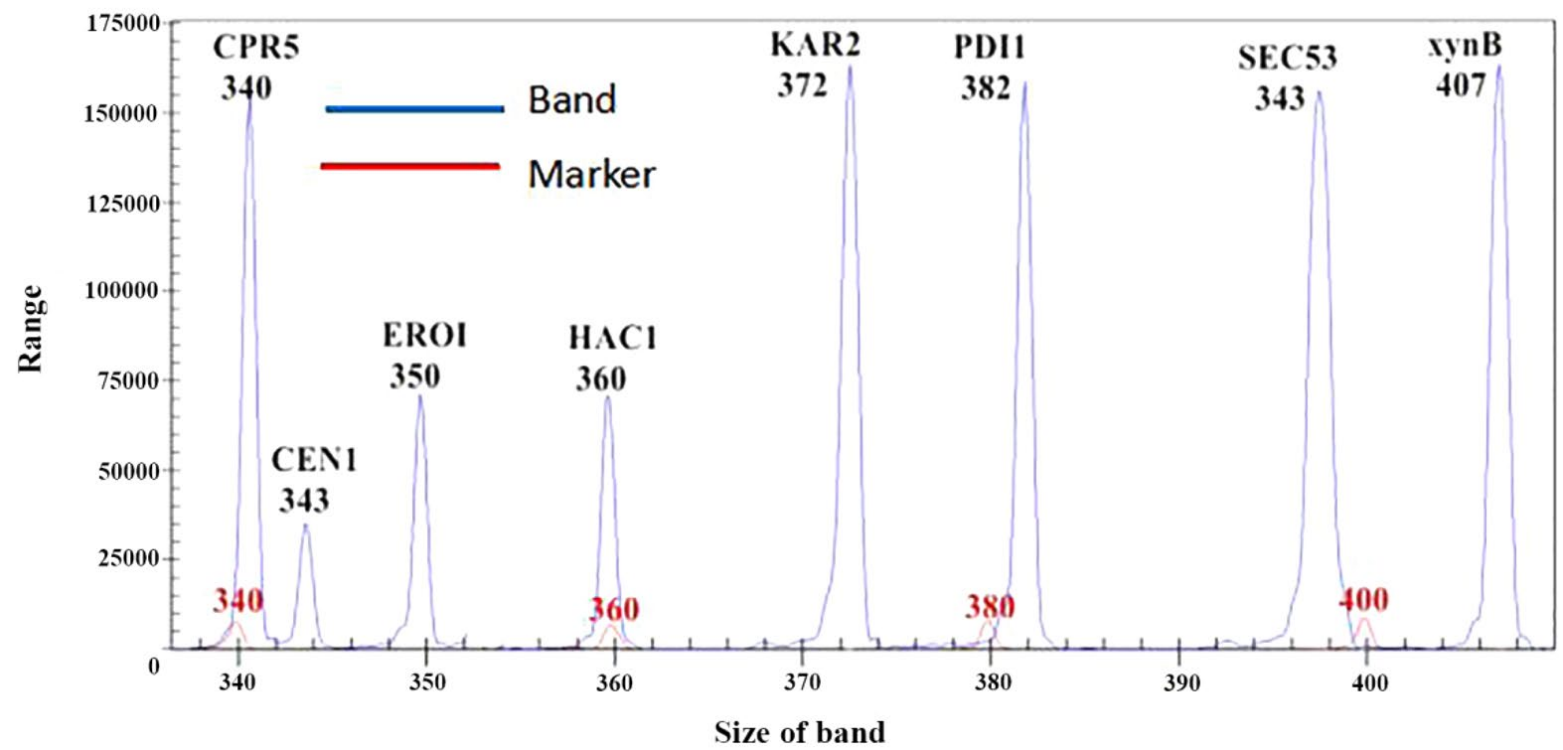
Electropherogram of reactions containing mixed primers. The red line indicates the size of the maker band (bp), while the blue line indicates the size of the target gene (bp).

In the GeXP electrophoretogram, the sequence size was indicated on the abscissa, while the size of the test fragment was determined based on the marker sizes. cDNA from the 24-h S8-H culture was used as the template. Primers targeting *CPR5*, *CNE1*, *ERO1*, *KAR2*, *HAC1*, *PDI1*, *SEC53* and *xynB* were mixed in equal proportions to prepare the reaction mix, and GeXP-based analysis was conducted to investigate primer specificity ( Fig. 4). The results showed good amplification of all eight genes, which could be used in subsequent experiments, and confirmed that the primers, general labels and fluorescent labels worked effectively.

#### *Effects of overexpression of* HAC1 *on genes associated with protein folding in the ER in strains S0 and S8*

To investigate the effects of overexpression of *HAC1* on the expression of protein folding-associated genes in wild-type strain S0, the expression of target genes in strains S0 and S0-H at 24 h, 48 h and 72 h post-inoculation were examined using the GeXP system. The results for the two strains over the same time period were compared using GeXP data analysis (Fig. S2A–C). Because *xynB* was not present in either S0 or S0-H, xylanase protein was not expressed and the band corresponding to the 407-bp *xynB* gene was absent.

As shown in Fig. S2A, the expression levels of all protein folding-associated genes apart from *KAR2* and *HAC1* were higher in strain S0 than in S0-H at 24 h post-inoculation. This may be because at 24 h, the strains were undergoing exponential growth and needed a good supply of proteins, with the ER capable of meeting the requirements of protein folding and assembly. Therefore, the UPR mechanism was not needed. It is also possible that the expression of *HAC1*, introduced on the vector, lagged behind that of native gene expression, meaning that *HAC1* was not fully expressed. At 48 h post-inoculation, the expression of seven protein folding-associated genes in S0-H was higher than that in S0 (Fig. S2B and C), indicative of an accumulation of secondary metabolites and the overexpression of *HAC1*, resulting in increased expression of other protein-folding associated genes in S0-H. Preliminary studies showed that overexpression of *HAC1* significantly elevated the expression of *xynB* in strain S8. Further investigation of the effects of overexpression of *HAC1* on the expression of ER protein folding-associated genes in strain S8 confirmed that overexpression of *HAC1* increased the expression of heterologous protein. The expression levels of the various genes in strains S8 and S8-H were then examined at 24 h, 48 h and 72 h post-inoculation and analysed using the GeXP system. As shown in Fig. S3A–C, the expression of all protein folding-associated genes in strain S8-H was higher than that in strain S8 at each time point, but was particularly noticeable at 48 h post-inoculation. The greatest differences in expression between the two strains were observed for *HAC1*, *SEC53* and *PDI1*.

Although strain S8 showed the highest level of xylanase expression among the high copy number strains, the overexpression of *HAC1* in strain S8-H significantly increased the expression of *CPR5*, *CEN1*, *ERO1*, *KAR2*, *PDI1* and *SEC53* compared with that in strain S8. It is possible that the overexpression of *HAC1* increased the involvement of the proteins encoded by the tested genes in the process of protein folding and assembly, ensuring proper folding and assembly of protein in the ER. In addition, the expression of *xynB* in strain S8-H was higher than that in strain S0 across all time points, which provides evidence that the overexpression of *HAC1* could further improve the expression of *xynB*.

#### Qualitative and quantitative analysis of protein folding-associated genes in strains S0-H, S1-H, S8-H and S22-H

**Figure 5:**
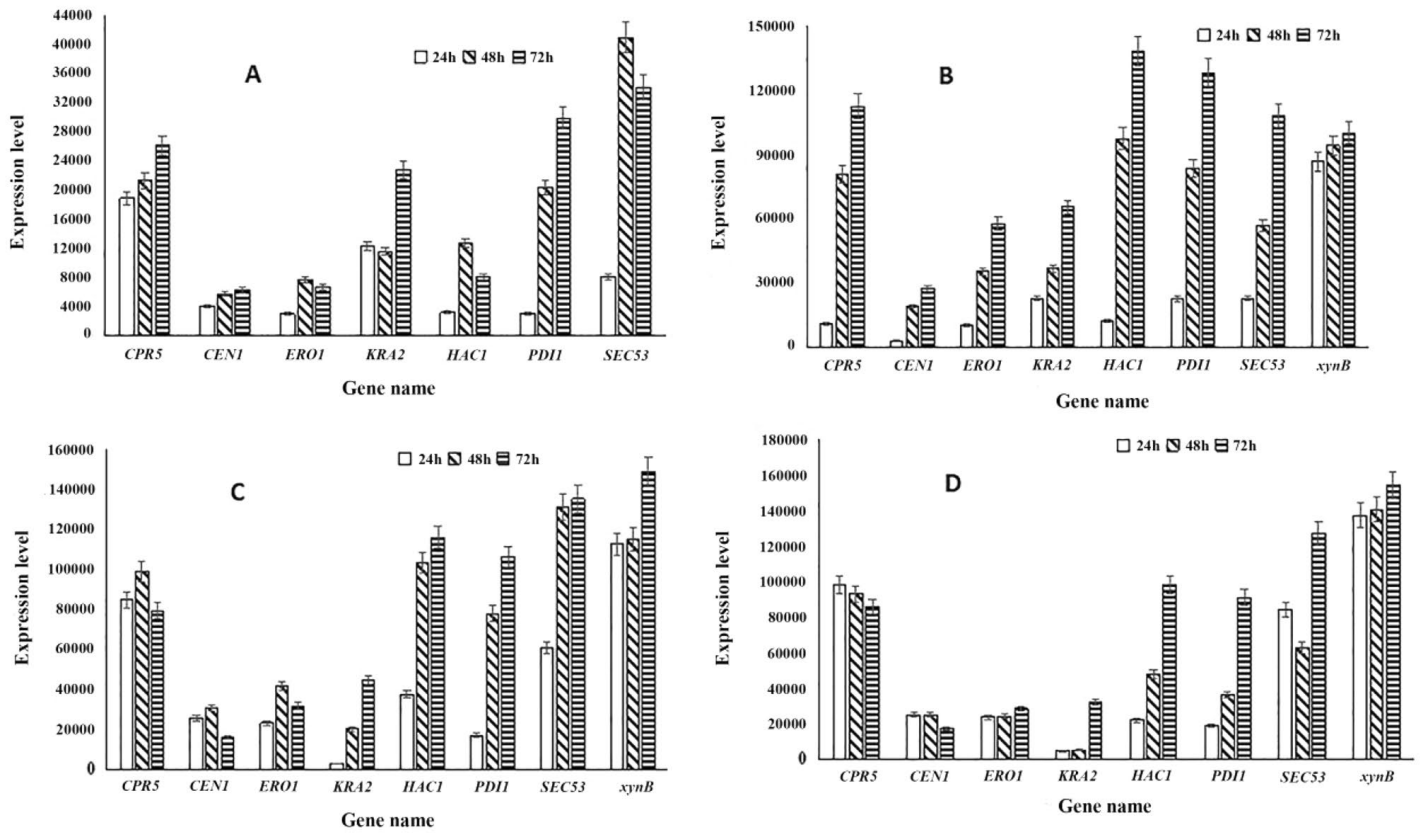
Quantitative analysis of protein folding-associated genes in strains S0-H (**A**), S1-H (**B**), S8-H (**C**) and S22-H (**D**).

The sequential changes in expression of genes associated with ER protein folding following overexpression of *HAC1* in *S. cerevisiae* strain S0 are shown in Fig. S4. The GeXP quantitative analysis tool was then used to quantitatively assess the expression levels of each of the genes at different time points (Fig. 5A). Although strain S0 did not express xylanase protein, the overexpression of *HAC1* triggered changes in a series of genes associated with protein folding, which indirectly indicated that Hac1p regulated the expression of these genes. As shown in Fig. S4 and Fig. 5A, the expression of genes associated with protein folding involved a series of sequential changes; specifically, the expression of each gene increased over time. Among the tested genes, the greatest increases in expression were observed for protein disulphide isomerase-encoding gene *PDI1* and phosphomannose-encoding gene *SEC53*. Compared with the levels at 24 h post-inoculation, the expression levels of *PDI1* and *SEC53* at 48 h and 72 h post-inoculation were increased by 5.51-fold and 8.54-fold and 4.04-fold and 3.19-fold, respectively. In comparison, the expression of *HAC1* at 72 h post-inoculation decreased by 36.3% compared with that at 48 h, suggesting higher levels of protein synthesis at 48 h post-inoculation than at 72 h, and that *HAC1* may only be partially expressed at the later time point.

The results of GeXP analysis of gene expression in single-copy *xynB* strain S1-H are shown in Fig. S5 and Fig. 5B. Compared with strain S0-H, the expression levels of all protein folding-associated genes were significantly increased, suggesting that they all participated in the expression of xylanase. At 72 h post-inoculation, the expression of cyclophilin protein-encoding gene *CPR5*, protein disulphide isomerase-encoding gene *ERO1* and calcium binding protein-encoding gene *CNE1* was decreased compared with that at 48 h. This may indicate that the single-copy expression of xylanase did not cause stress on the ER, and that basal levels of expression of the protein folding-associated genes were sufficient to cover the needs of protein folding and assembly. In addition, the expression of *xynB* reached a maximum at 72 h post-inoculation, which was consistent with the enzyme activity results showing that enzyme activity reached a maximum at 72 h.

Strain S8 had the highest level of xylanase expression. However, overexpression of *HAC1* further increased expression levels, suggesting that overexpression of *HAC1* may enhance the capacity of the cells to process heterologous proteins. Gene expression was then examined in strain S8-H at 24 h, 48 h and 72 h post-inoculation using the GeXP system (Fig. S6 and Fig. 5C). Results showed that overexpression of *HAC1* resulted in increased expression of all protein folding-associated genes except *CPR5*, the protein product of which participates in post-translational modification of proteins, and *CEN1*, whose corresponding protein is involved in glycosylation modification of proteins. At 72 h post-inoculation, the expression of *CPR5* and *CEN1* was decreased, which may be associated with the hysteresis effect of protein synthesis and secretion, or with the activation of the UPR by *HAC1* expression, which triggers protein degradation and a series of changes within the cell. These results suggested that the expression and secretion of proteins in *S. cerevisiae* is a complicated process. At 72 h post-inoculation, the expression of *HAC1* was 4.09-fold and 2.03-fold higher than that at 24 h and 48 h, respectively. Accordingly, it might be possible that in addition to the activated *HAC1* gene, the endogenous *S. cerevisiae HAC1* gene was also expressed. Further investigation showed that *xynB* expression at the three different time points resulted in similar patterns of enzyme production, reaching 154,265 at 72 h post-inoculation, which was the highest protein yield among all strains. This finding confirmed that eight copies of *xynB* resulted in the highest xylanase productivity.

Although strain S22 had the highest *xynB* copy number, this did not correlate with the highest levels of xylanase expression. Gene expression levels in strain S8-H at 24 h, 48 h and 72 h post-inoculation were investigated using the GeXP system, and results are shown in Fig. S7 and Fig. 5D. The expression levels of protein folding-associated genes were significantly increased at 48 h and 72 h post-inoculation compared with levels in strain S22. However, at 24 h post-inoculation, gene expression levels of strain S22-H were lower than those of strain S1-H. The high gene copy number in strain S22-H could lead to high levels of the xylanase protein, causing cellular stress and resulting in retarded growth in the early stages of cultivation. Moreover, compared with the 24-h and 48-h time points, gene expression levels in strain S22-H were increased at 72 h post-inoculation. This suggested that at 72 h post-inoculation, the synthesis of heterologous proteins required the participation of protein folding and assembly genes, which was consistent with the results of xylanase gene and enzyme production analyses for this strain.

## Discussion

In the current study, high-yield xylanase-producing *S. cerevisiae* strains were generated via promoter modification, the introduction of secretory signal peptides and increasing the gene copy number. Analysis showed that the relationship between xylanase gene copy number and enzyme activity was not linear, with more than eight copies of *xynB* shown to be detrimental to protein expression. These findings suggest that the protein folding function of the ER limits further improvement of enzyme activity. However, overexpression of pathway transcription factor gene *HAC1* significantly increased the expression of genes involved in ER protein folding and processing, resulting in high levels of xylanase expression.

### Constitutive secretory expression of xylanase via the *PGK1p* and α-factor signal peptide

The promoter is one of the most crucial elements of an expression vector because it influences the mRNA synthesis rate. Accordingly, a strong promoter contributes to higher expression of a heterologous protein^19^. To improve the expression of cellobiohydrolase in *A. niger*, Qin et al. used the *glaA* promoter sequence, which promotes the expression of glucoamylase, to drive the expression of the *CBH* gene. Enzyme activity testing showed that the filter paper activity of the resulting recombinant strains was 2.51-fold higher than that of the wild-type strain^20^. In the current study, the galactose-inducible promoter of the vector was replaced with the constitutive *PGK1p* sequence, allowing direct expression of xylanase in YPD medium and significantly improving expression in the recombinant strains.

Signal peptides are short peptides that guide the secretion of newly-synthesised heterologous proteins and improve their solubility, making them the focus of many studies in recent years^21,22^. Sodium dodecyl sulphate polyacrylamide gel electrophoresis analysis has shown that the introduction of a signal peptide improves the secretion of heterologous proteins. In the current study, the preferred *S. cerevisiae* signal peptide, from α-factor, was used to construct a strain in which xylanase was successfully excreted.

### Improvement of xylanase expression by the introduction of multiple gene copies

Gene copy number affects the expression level of heterologous proteins. Accordingly, increasing the number of copies of a target gene can effectively increase the heterologous protein yield. Jeong et al. showed that an increased gene copy number improved the production of tripeptide His-His-Leu compared with the single gene copy strain, and found that the protein was more stable^23^. Tamakawa et al. developed a series of *Candida utilis* strains containing 1–10 copies of genes *mXYL1*, *XYL2* and *XYL3*, encoding mutated xylose reductase K275R/N277D, C-xylitol dehydrogenase and xylulokinase, respectively, to examine the relationship between gene expression and copy number. Their results showed that there was a positive but non-multiple relationship between the two variables^24^. Another study showed that for secreted proteins, copy number and protein expression levels were positively correlated within a certain range, with four to nine gene copies being optimal and additional copies resulting in decreased protein expression in *Pichia pastoris*^25^. In the current study, the xylanase activity of transformants containing different copy numbers of *xynB* was examined. The results showed that eight gene copies resulted in maximum xylanase activity, but that when the copy number exceeded eight, xylanase activity decreased. These results confirmed that increasing the gene copy number does not necessarily increase protein expression.

### Overexpression of *HAC1* enhances the expression of heterologous proteins

Hac1p, the transcriptional regulator of the UPR pathway, interacts with a large number of molecular chaperones associated with protein folding and assembly. The UPR mechanism in eukaryotic expression systems has been a hot topic in recent years, with much research focused on improving the expression of heterologous proteins, as well as genes associated with protein folding in the ER, by regulating *HAC1* expression^26,27^. Lee et al. studied the effects of overexpression of a UPR pathway gene in recombinant *S. cerevisiae* strains harbouring 16 copies of the gene coding for human kringle protein LK8. Their results showed that Hac1p promotes LK8 protein production as well as cell growth in recombinant *S. cerevisiae*^28^. In the current study, *HAC1* was overexpressed in recombinant *S. cerevisiae* strains containing one, eight (the highest enzyme activity), or 22 (the highest number of copies) copies of *xynB*, with overexpression of *HAC1* in the wild-type strain used as a control. The expression levels of protein folding-associated genes with and without *HAC1* overexpression were then investigated using the GeXP system. The results showed that even in the wild-type (S0) strain, overexpression of *HAC1* enhanced the expression of protein folding-associated genes. In addition, compared with strains S8 and S22, the gene expression levels in strains S8-H and S22-H were significantly increased, indicating that eight and 22 copies of *xynB* caused stress in the ER.

In *S. cerevisiae* expression systems, the production and accumulation of misfolded proteins, especially the overexpression of proteins from multiple copies of a gene, are key to the efficient expression of heterologous proteins^29^. These proteins cannot be properly folded, so they are accumulated in the ER, triggering the ER-associated protein degradation (ERAD) pathway, resulting in degradation by ER protease. The current investigation of the expression levels of genes associated with protein folding in strains S8-H and S22-H showed that in high-copy strains, the sequential changes in gene expression levels were not as regular as those in low-copy and wild-type strains. It is therefore likely that this is caused by the effects of the ERAD pathway on gene expression.

In summary, by applying rDNA integration, multi-copy constitutive secretory expression of xylanase was successfully achieved in *S. cerevisiae*. Strain S8, with eight copies of *xynB*, showed the highest levels of xylanase expression, reaching a final yield of 325 U/mL. However, overexpression of *HAC1* increased the xylanase production capacity of strain S8, resulting in a final yield of 381 U/mL. Overall, this study revealed that overexpression of *HAC1* enhances the expression of genes associated with protein folding in the ER and improves the protein folding functionality of the ER, which in turn further enhances the expression of xylanase.

## Supplementary information

Supplementary file1 (DOCX 7825 kb)

## Data Availability

All data generated or analysed during this study are included in this published article (and its Supplementary Information files).
