# Supplementary material for: Expression and function of an Hac1-regulated multi-copy xylanase gene in Saccharomyces cerevisiae
Source: Sci Rep. 2020 Jul 15;10:11686. doi: 10.1038/s41598-020-68570-6 (PMC7363925; doi:10.1038/s41598-020-68570-6)
Supplement: Supplementary file 1 — Supplementary file1 (DOCX 7825 kb) [file 41598_2020_68570_MOESM1_ESM.docx]

**Expression and function of an *Hac1*-regulated multi-copy xylanase gene in**

***Saccharomyces cerevisiae***

Changjie Bao^a,b,1^, Jiping Li^c,1^, Huan Chen^a,b^, Yang Sun^a,b^, Gang Wang^a,b^, Guang Chen^a,b^,Sitong Zhang^a,b,*^

^a^College of Life Sciences, Jilin Agricultural University, Changchun, China

^b^Key Laboratory of Straw Biology and Utilization, The Ministry of Education, Changchun, China

*^c^Institute of Antler Science and Product Technology, Changchun Sci-Tech University, Changchun, China*

*^*^*The corresponding author. E-mail:18943132269@163.com (S. Zhang)

^1^These authors contributed equally to this work and should be considered co-first authors.

**The following is a list of authors email or a link to their publication record(s)**

1. Changjie Bao:bao875472264@163.com
2. Jipng Li:1152853345@qq.com
3. Huan Chen:<http://www.sciencedirect.com/science/article/pii/S0960852417305965>
4. Yang Sun:<http://www.sciencedirect.com/science/article/pii/S0960852417305965>
5. Gang Wang:[gawang@ucdavis.edu](mailto:gawang@ucdavis.edu)
6. Guang Chen:chg61@163.com
7. Sitong Zhang:18943132269@163.com

**List of Abbreviations**

| **Abbreviations** | **Full name/Paraphrase** |
| --- | --- |
| ddPCR | Droplet digital polymerase chain reaction |
| CNE1 | a calcium-binding protein, acts as a molecular chaperone in the ER and participates in protein folding and glycosylation modification |
| CPR5 | is a cyclophilin, the main functions of which are to affect peptidyl-prolyl *cis*-*trans* isomerase, participate in post-translational modification of proteins, and maintain the homeostasis of intracellular metal ions |
| *CYC1T* | *CYC1* terminator |
| ER | endoplasmic reticulum |
| ERO1 | PDI oxidase, controls the folding of oxidized proteins in the ER |
| GeXP | Genetic Analysis System |
| HAC1 | Transcriptional activator HAC1 |
| *HAC1* | Transcriptional activator HAC1 gene |
| KAR2 | a Bip-binding protein that transports proteins to the ER with the help of an ATPase and also acts as a molecular chaperone |
| PCR | polymerase chain reaction |
| PDI1 | a multifunctional molecular chaperone in the ER and plays a major role in the formation of disulfide bonds |
| *PGK1* | Phosphoglycerate kinase gene |
| rDNA | Ribosomal DNA |
| RT-PCR | reverse-transcriptase PCR |
| *S. cerevisiae* | *Saccharomyces cerevisiae* |
| SEC53 | a phosphomannomutase, is involved in the synthesis of GDP-mannose and mannose 6-phosphate, which are required for the folding and glycosylation of secretory proteins in the ER lumen |
| UPR | unfolded protein response |
| *xynB* | β-1,4-endoxylanase B |
| α-factor | the α-factor signal peptide |

Note: Arrange by the alphabetical order of capital letters

Fig. S1 Detection of xynB copy number in *S. cerevisiae* INVSc1[pYES2*-*PαXC*-*rDNA] transformants by ddPCR


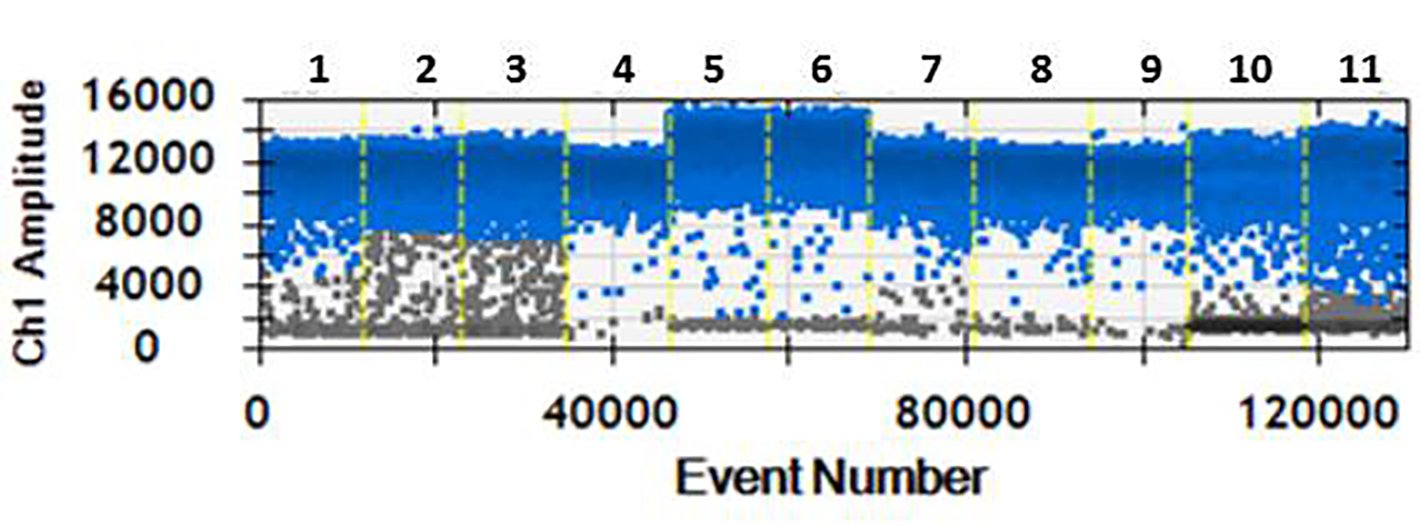


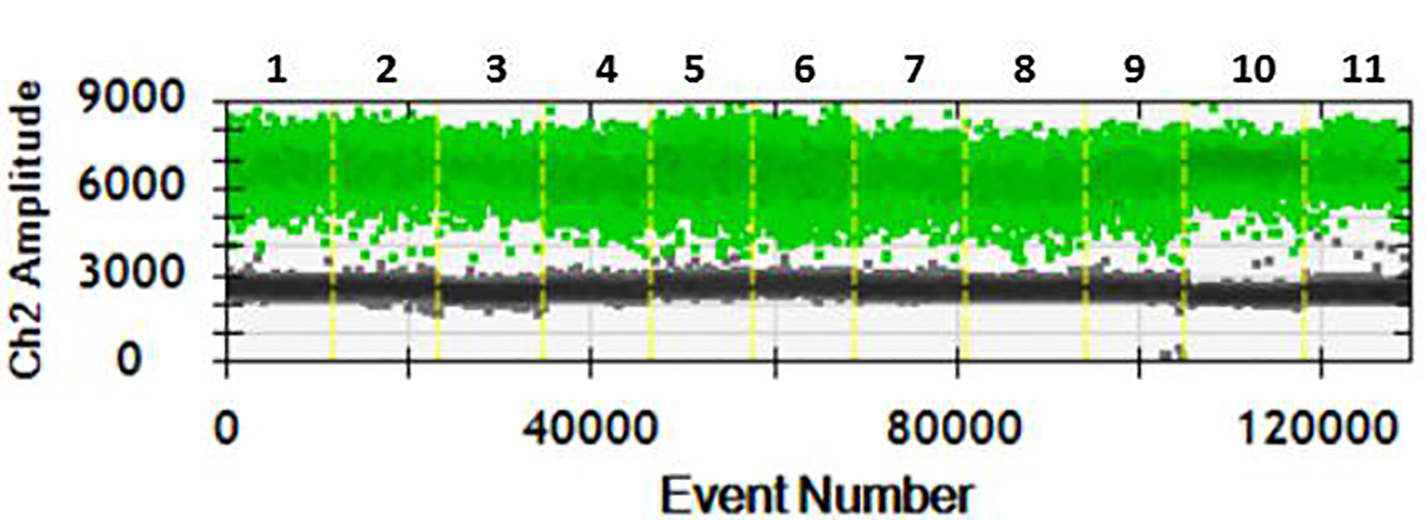


**Fig. S2 Comparative analysis of protein folding-associated genes in strains S0 and S0-H at 24 h (A), 48 h (B), and 72 h (C) post-inoculation**

**
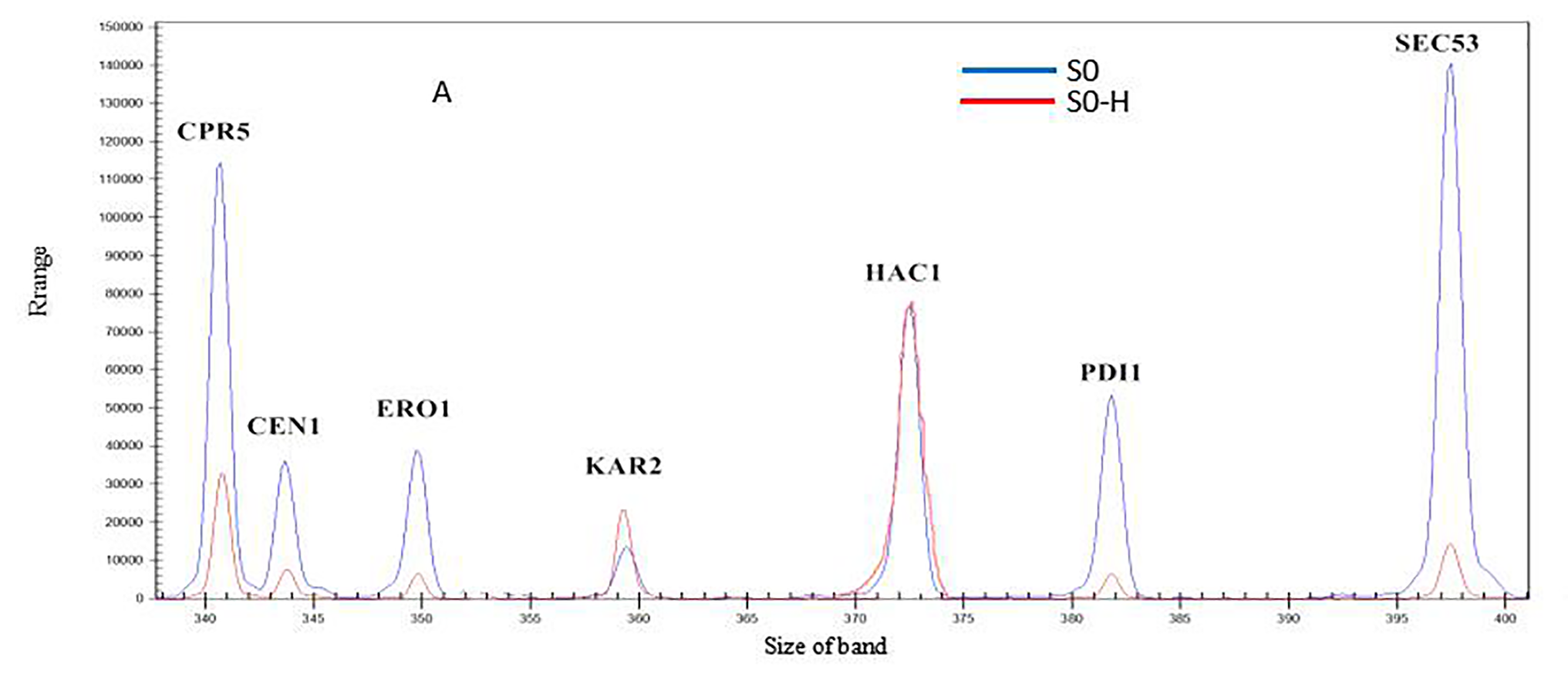
**

**
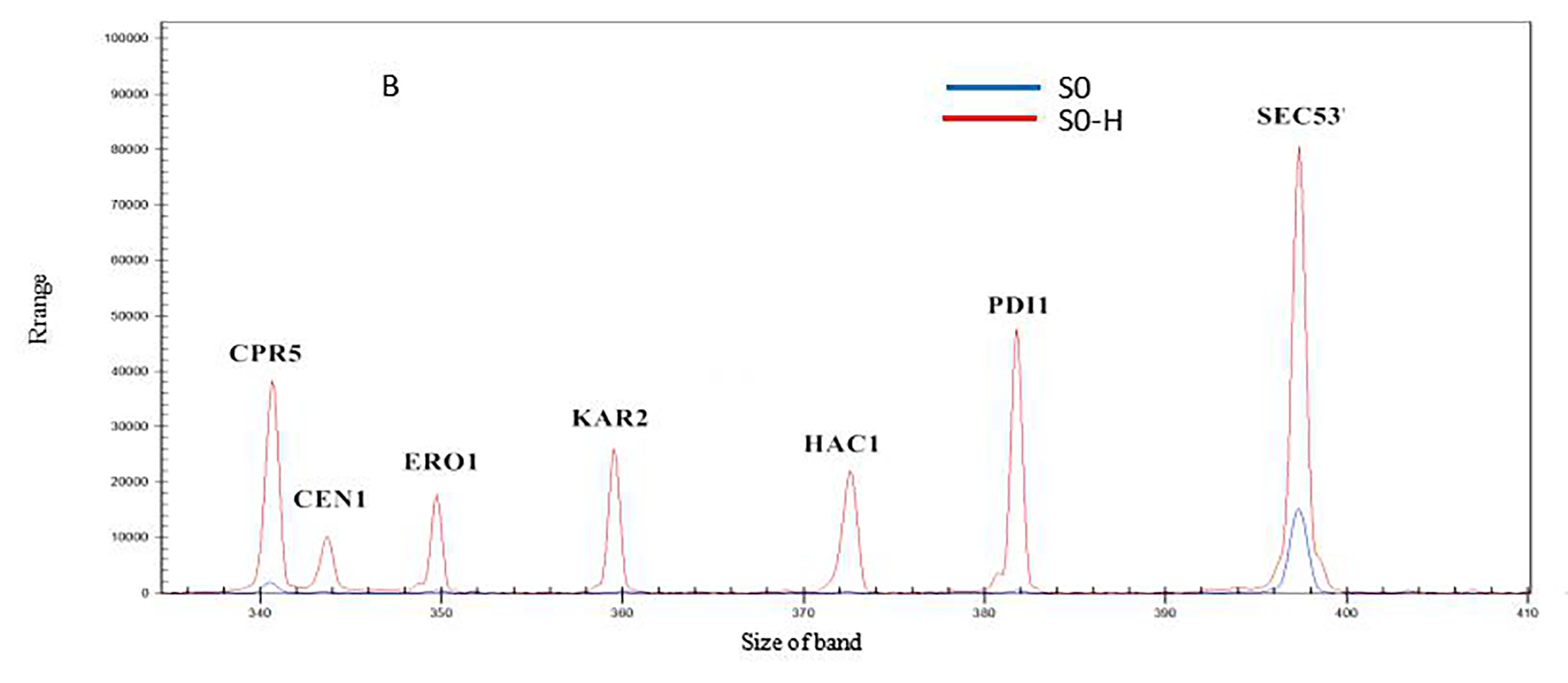
**

**
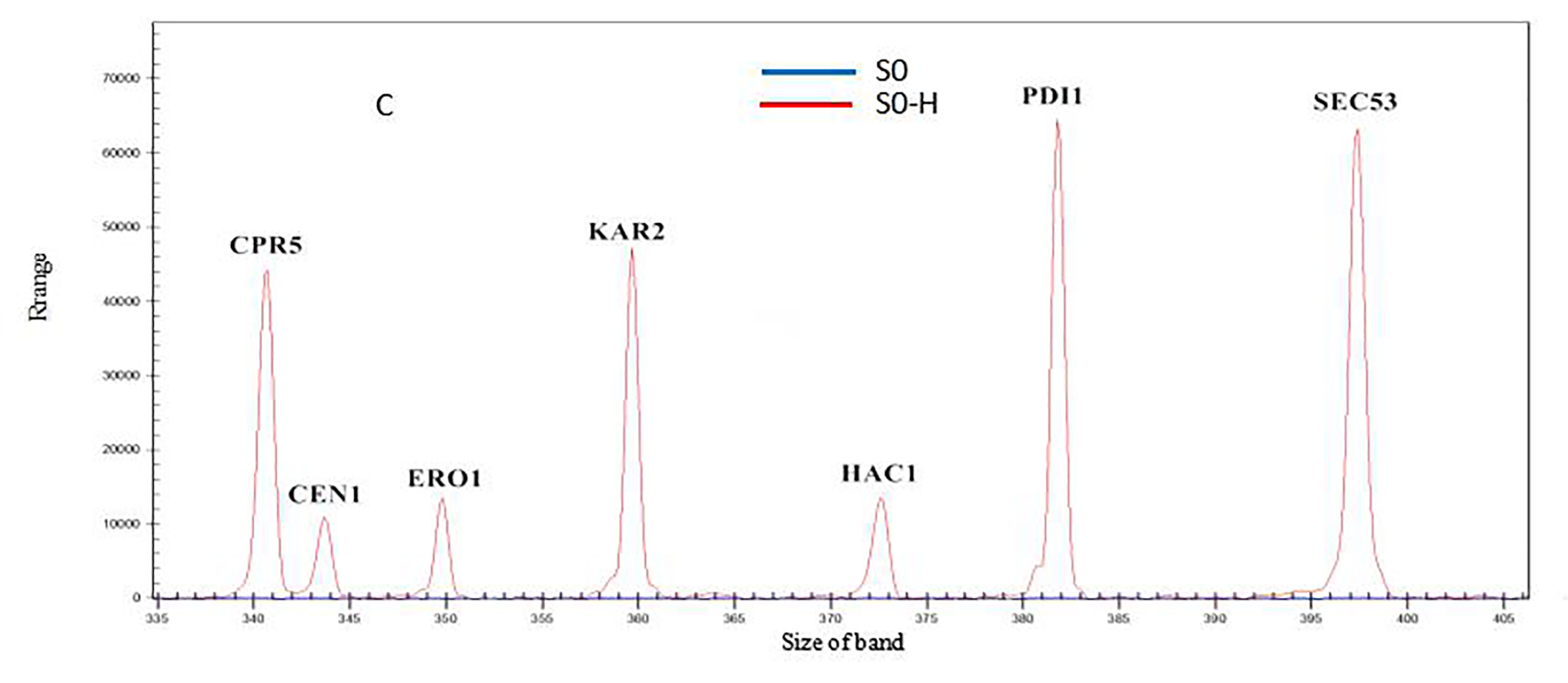
**

**Fig. S3 Comparative analysis of protein folding-associated genes in strains S8 and S8-H at 24 h (A), 48 h (B), and 72 h (C) post-inoculation**


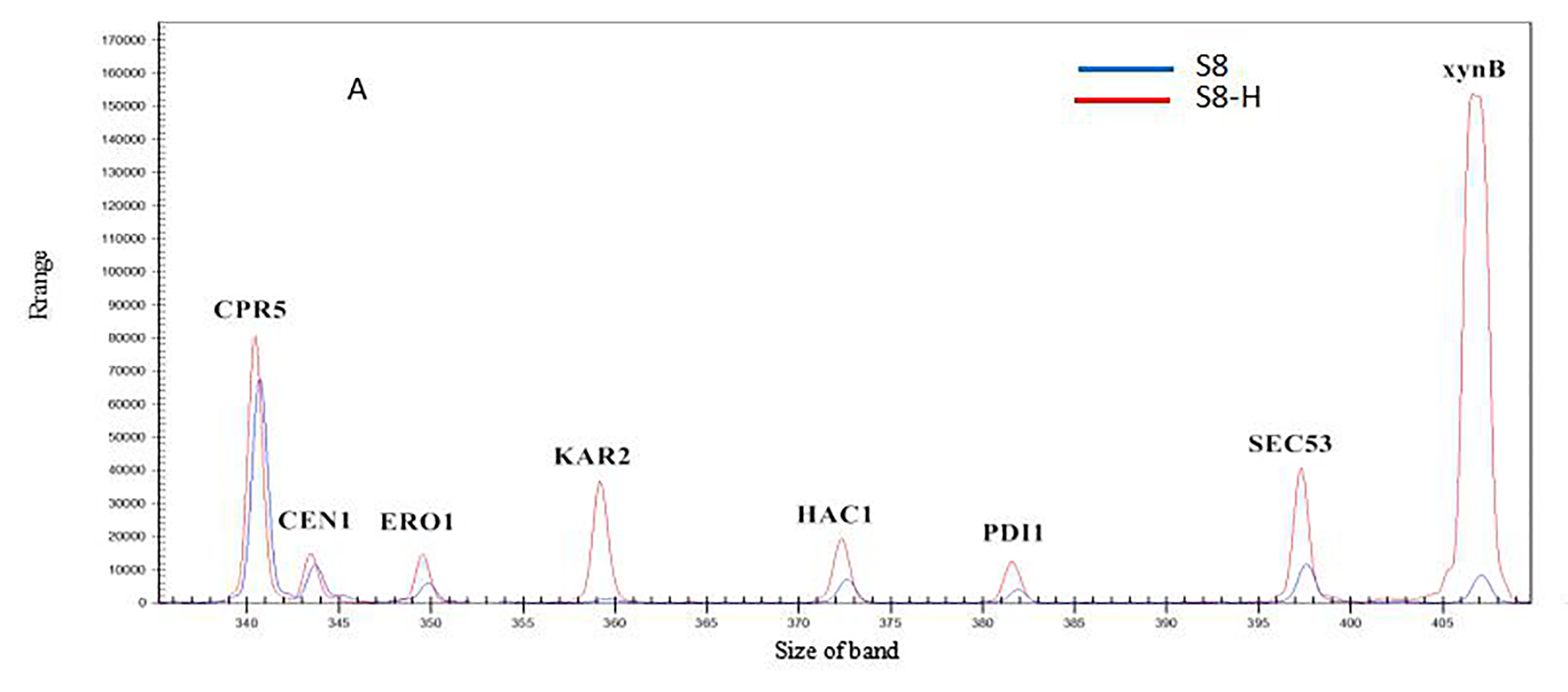


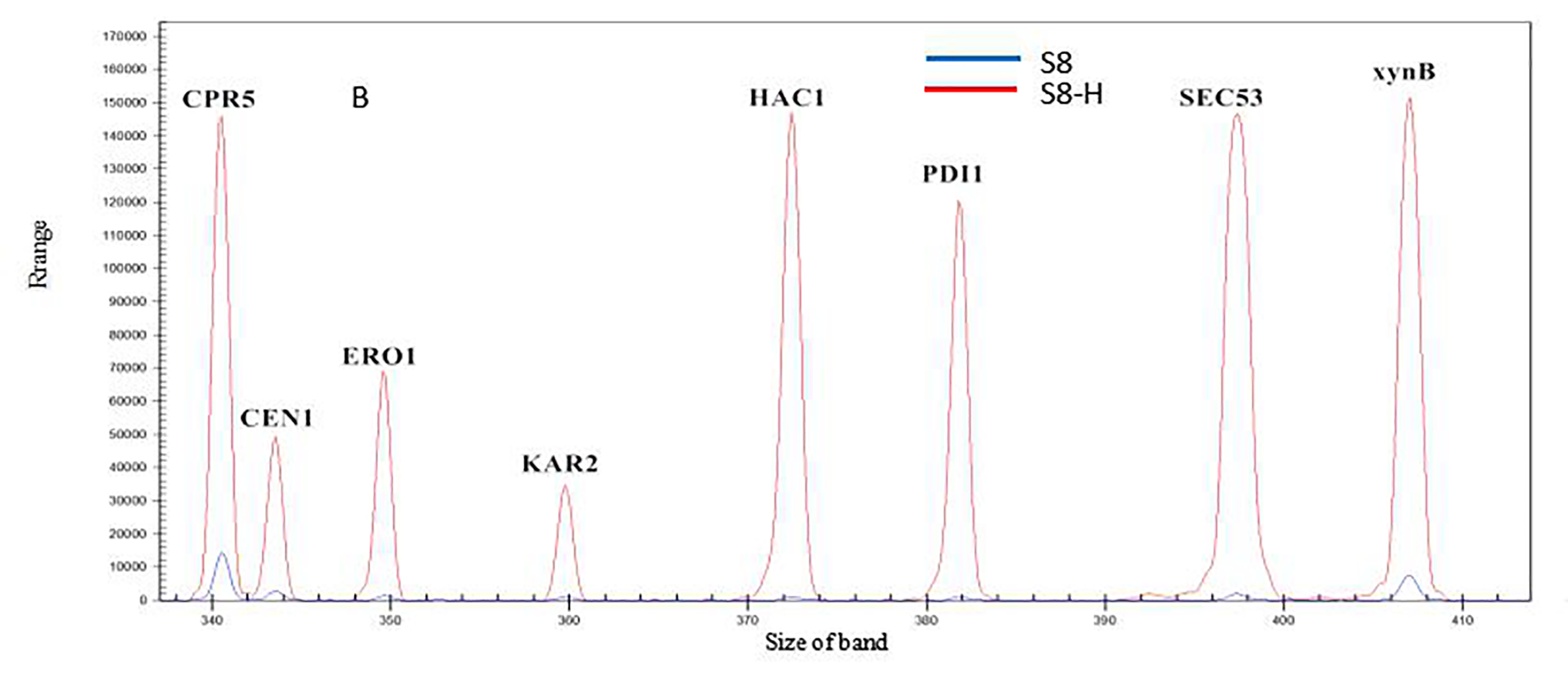


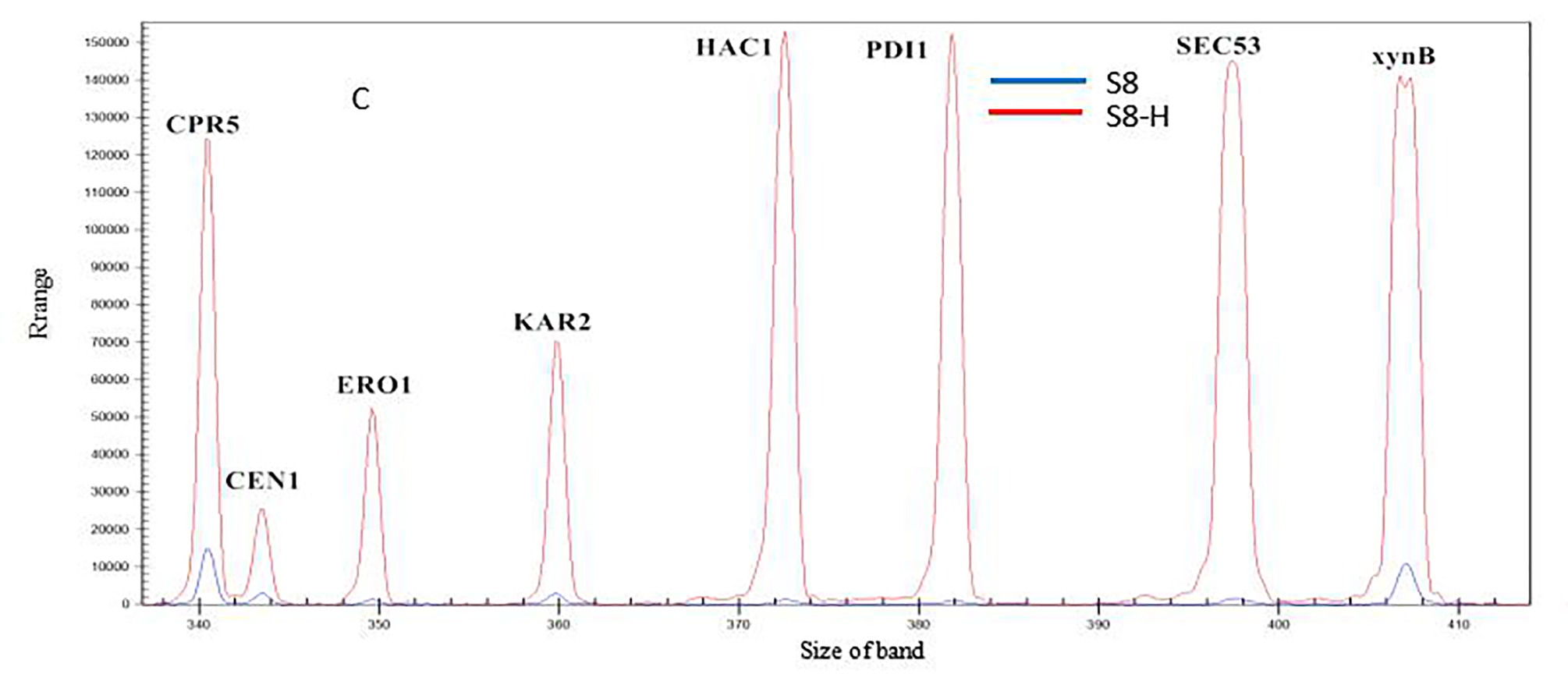


**Fig. S4 Sequential changes in protein folding-associated genes in strain S0-H**

**
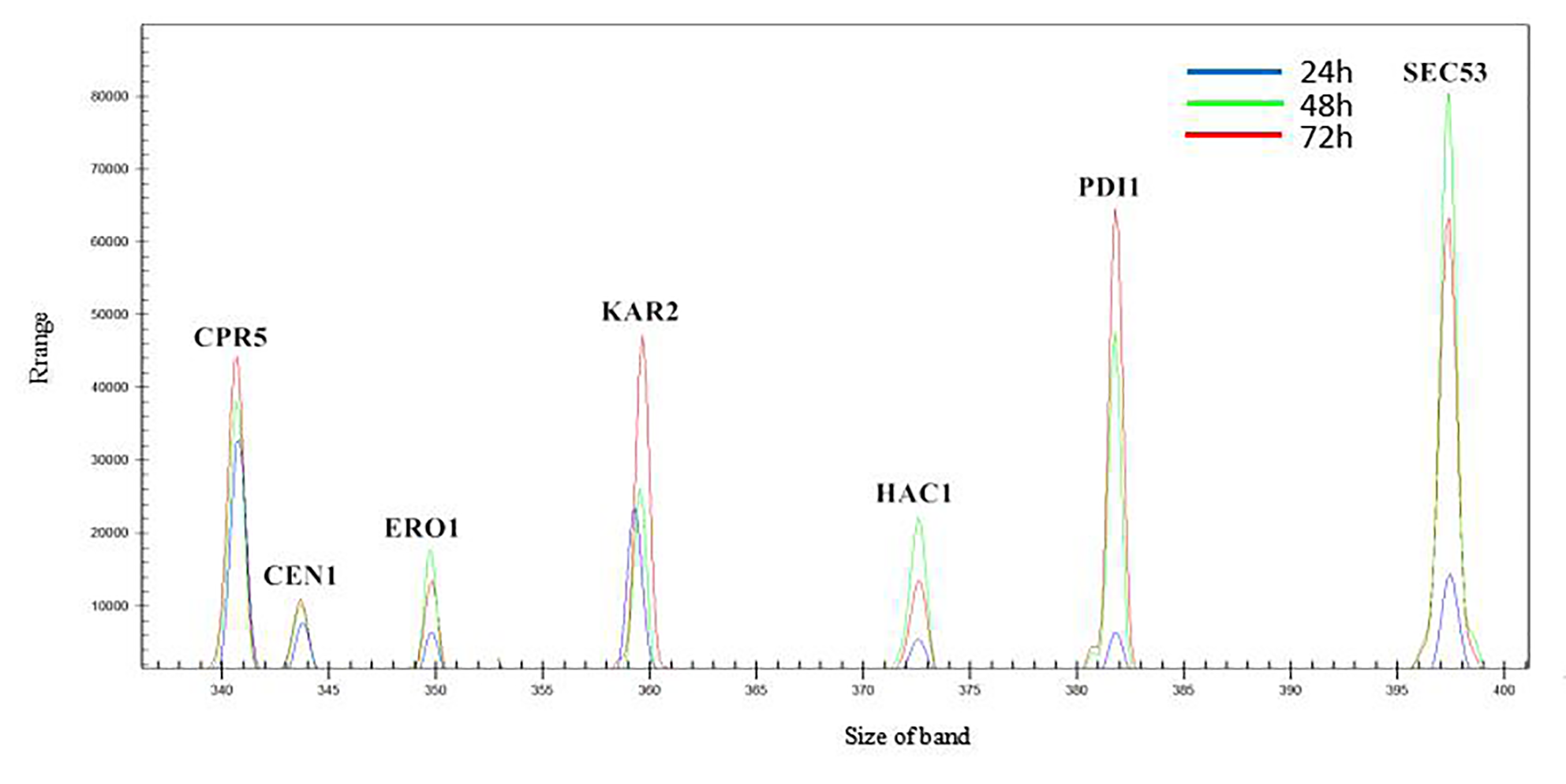
**

**Fig. S5 Sequential changes in protein folding-associated genes in strain S1-H**

**
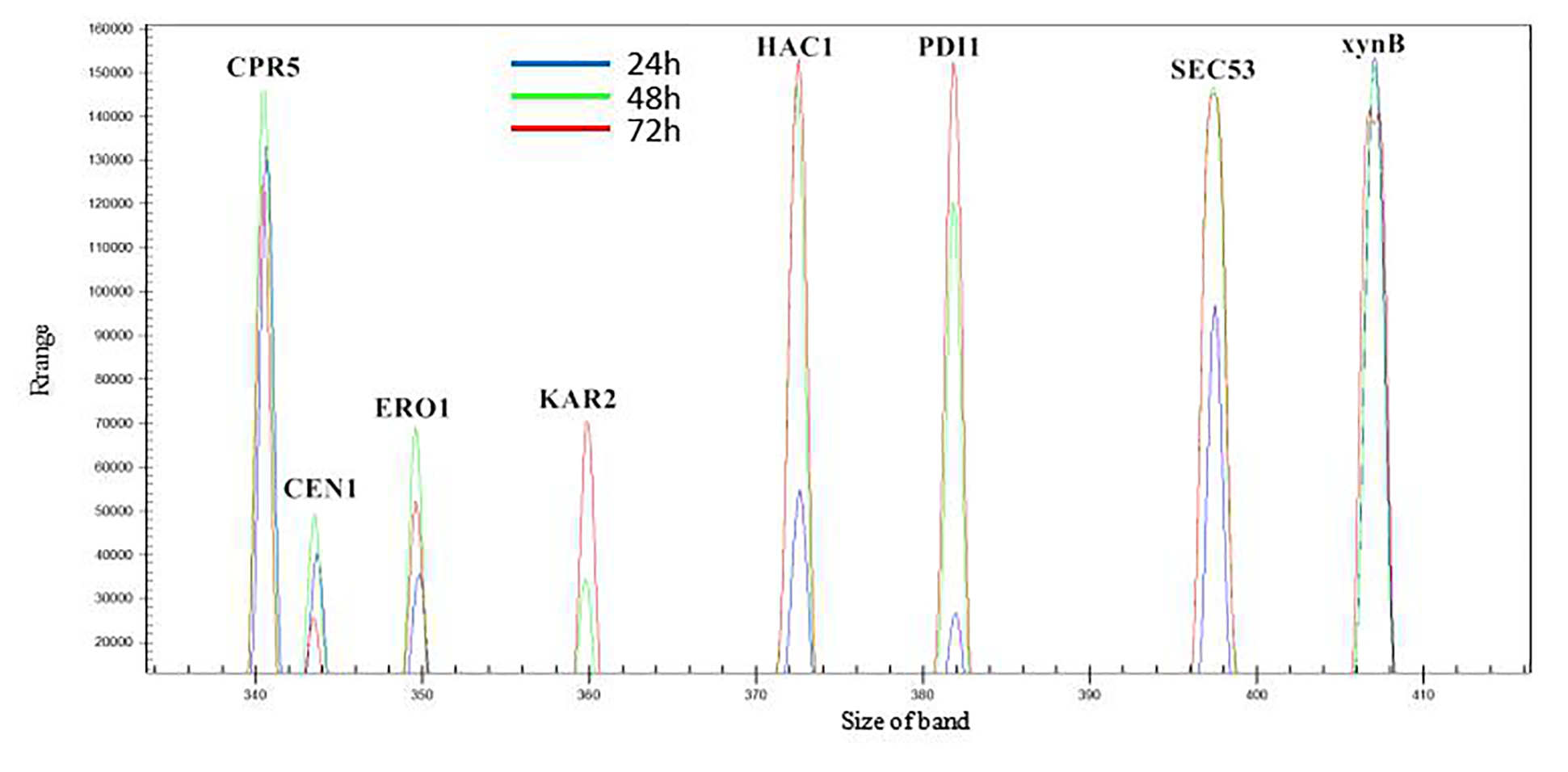
**

**Fig. S6 Sequential changes in protein folding-associated genes in strain S8-H**

**
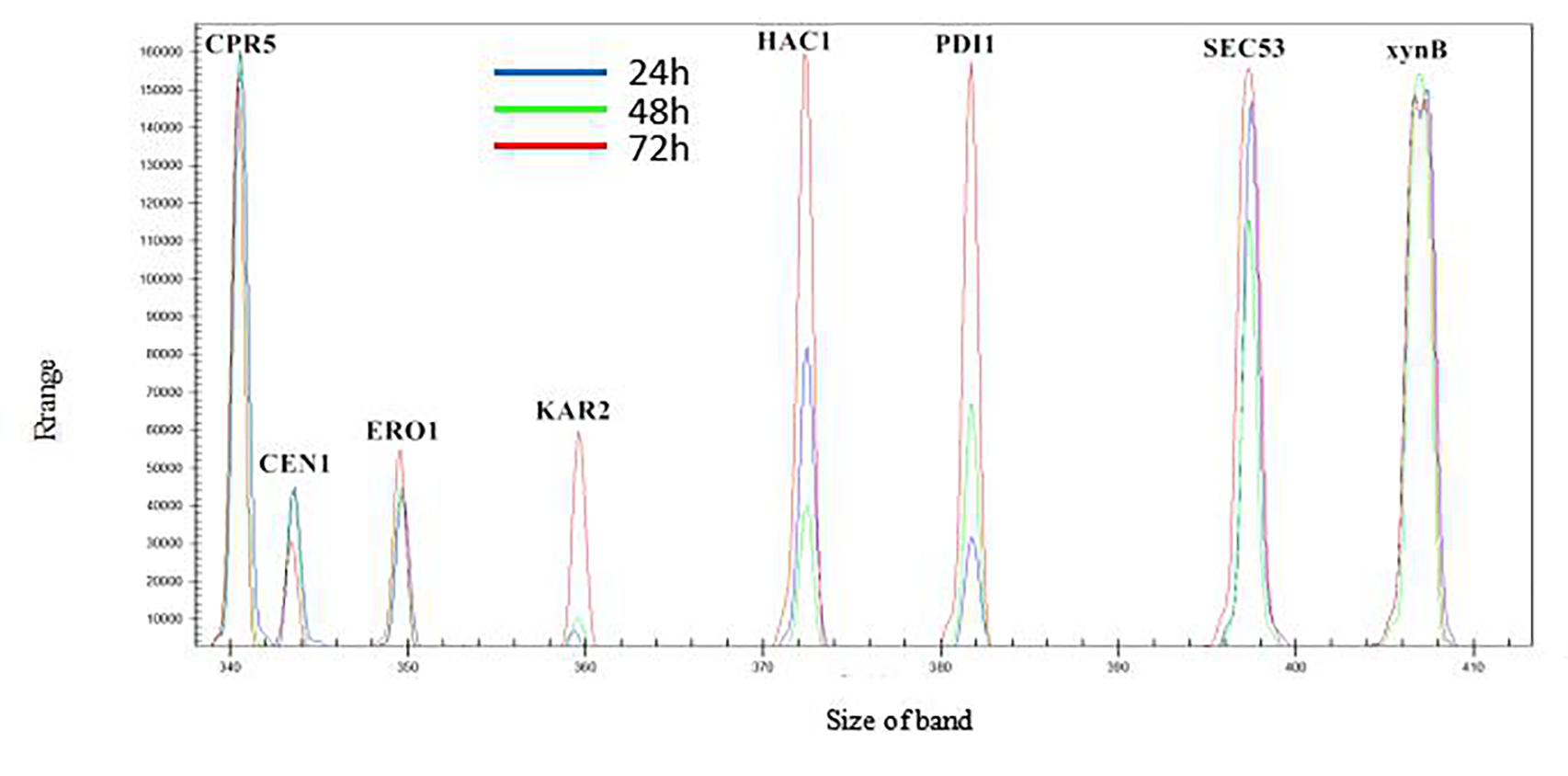
**

**Fig. S7 Comparative analysis of protein folding-associated genes in strains S22 and S22-H**

**
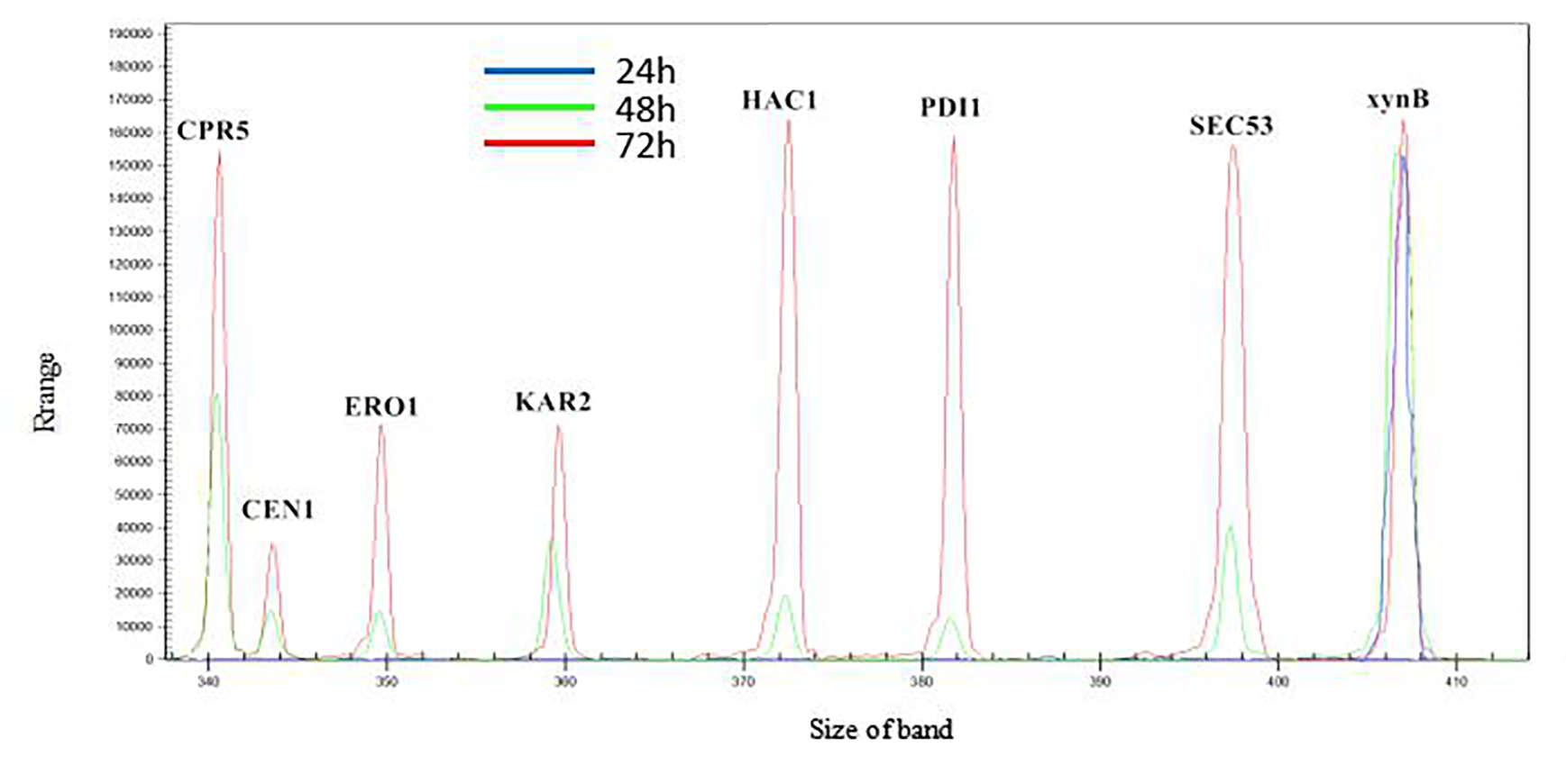
**
